# Efficient seed-mediated method for the large-scale synthesis of Au nanorods

**DOI:** 10.1007/s11051-017-3815-9

**Published:** 2017-03-17

**Authors:** Waqqar Ahmed, Arshad Saleem Bhatti, Jan M. van Ruitenbeek

**Affiliations:** 1 Department of Physics, COMSATS Institute of Information Technology, Park Road, Islamabad, 44000 Pakistan; 2 Huygens-Kamerlingh Onnes Laboratory, Leiden University, Niels Bohrweg 2, 2333 CA Leiden, The Netherlands

**Keywords:** Gold nanoparticles, Seeded protocol, Large-scale synthesis, Plasmonics

## Abstract

Seed-mediated methods are widely followed for the synthesis of Au nanorods (NRs). However, mostly dilute concentrations of the Au precursor (HAuCl_4_) are used in the growth solution, which leads to a low final concentration of NRs. Attempts of increasing the concentration of NRs by simply increasing the concentration of HAuCl_4_, other reagents in the growth solution and seeds lead to a faster growth kinetics which is not favourable for NR growth. Herein, we demonstrate that the increase in growth kinetics for high concentrations of reagents in growth solution can be neutralised by decreasing the pH of the solution. The synthesis of the NRs can be scaled up by using higher concentrations of reagents and adding an optimum concentration of HCl in the growth solution. The concentration of HAuCl_4_ in the growth solution can be increased up to 5 mM, and 10–20 times more NRs can be synthesised for the same reaction volume compared to that of the conventional seed-mediated method. We have also noticed that a cetyltrimethylammonium bromide (CTAB)-to-HAuCl_4_ molar ratio of 50 is sufficient for obtaining high yield of NRs.

## Introduction

The size- and shape-dependent optical properties of Au nanoparticles have made them a promising candidate for applications in sensing, photonics, bioimaging, biomedicine and nanoelectronics (Wang et al. 2010; Vigderman et al. 2012; Jiang et al. 2013; Dreaden et al. 2012; Ding et al. 2007; Huang et al. 2007; Huschka et al. 2011; Mohanta et al. 2016; Alvarez-Puebla et al. 2011; Stewart et al. 2014). Among various shapes, NRs are in high demand owing to the tunability and sensitivity of their longitudinal plasmon resonance. In order to meet the increasing demand, it is greatly desirable to develop a cost-effective and large-scale synthesis protocol for Au NRs.

Usually a surfactant-assisted and seed-mediated two-step method is followed for the Au NR synthesis. In the first step, Au seed particles are prepared, which are subsequently added to a growth solution. The growth solution usually contains an Au precursor (HAuCl_4_), growth directional agents (CTAB and AgNO_3_) and a reducing agent (ascorbic acid (AA)). However, in most of the reported protocols, dilute concentrations (0.25–0.5 mM) of HAuCl_4_ are used in the growth solution (Jana et al. 2001; Busbee et al. 2003; Nikoobakht and El-Sayed 2003; Johnson et al. 2002; Gole and Murphy 2004; Alekseeva et al. 2006; Busbee et al. 2003; Pérez-Juste et al. 2005; Johnson et al. 2002; Ye et al. 2013). Multiple batches of NRs, prepared even under identical conditions, do not necessarily provide NRs of the same dimensions and dispersion, which is often a drawback if large concentrations of identical NRs are needed e.g. for performing a set of experiments with identical NRs.

One may think that synthesis can be scaled up for a single batch simply by increasing the solution volume. However, simple scaling up leads to different thermal transports of reagents and mixing problems. Furthermore, for complete solubility of CTAB, the solution is usually heated to about 40–60 °C and then cooled down to room temperature. Heating and cooling of large solution volume require both extra energy and time.

Alternatively, the yield can also be scaled up by increasing the concentration of all the reactants in the growth solution. However, this will lead to a higher diffusion rate of the reagents. Consequently, much higher growth rates are expected which will ultimately lead to the formation of more isotropic NRs, i.e. NRs of lower aspect ratio.

Single-step, high-concentration synthesis of gold NRs has been reported by Jana (2005), but the yield of NRs was limited. Kou et al. also reported a single-step protocol for the synthesis of Au NRs of various aspect ratios using cetyltripropylammonium bromide and cetyltributylammonium bromide surfactants (2007). However, the yield in this case was lower as well. The lower yield in the single-step methods probably is a consequence of simultaneous nucleation and growth of Au and due to the high concentrations of HAuCl_4_ in the CTAB medium, which leads to the formation of insoluble Au-Br-CTA complexes. Seeded protocols are usually preferred over single-step methods, as the nucleation and growth steps are separated, which gives better control over growth kinetics and yields highly monodispersed NRs. Recently, for large-scale synthesis of Au NRs, microfluidic cells have also been employed with the use of either single-step or seeded protocols (Lohse et al. 2013; Duraiswamy and Khan 2009; Uson et al. 2016; Watt et al. 2015). While the synthesis with microfluidic cells is very elegant, we propose here a simpler seeded method for scaling up the synthesis.

We have investigated the effect of increasing Au concentration on the synthesis of Au NRs. The synthesis of NRs was carried out in a growth solution containing HAuCl_4_ concentration of up to 5 mM. We show that the increase in growth rate of NRs by increasing the reagent concentration in solution can be neutralised by decreasing the pH of the growth solution. By using an optimum value of the HCl concentration and the CATB-to-HAuCl_4_ molar ratio, monodispersed NRs of various aspect ratios having high yields (>90%) can be obtained. We have found that CTAB-to-HAuCl_4_ ratios of as low as 50 can be used for high-yield synthesis of Au NRs. This is about four times less than that of the conventional CTAB-to-HAuCl_4_ molar ratio of about 200 used for Au NR synthesis (Nikoobakht and El-Sayed 2003). As the bulk of the expenditure for Au NR synthesis in CTAB-assisted seeded protocols is due to CTAB (Xu et al. 2015), reduction of the CTAB-to-HAuCl_4_ molar ratio will also lead to a marked decrease in the cost of Au NRs.

## Experimental methods

### Synthesis of seeds

For the synthesis of seeds, 25 μl of 0.1 M HAuCl_4_ was added in 10 ml of 0.1 M CTAB solution. This was followed by the addition of 45 μl of 0.1 M NaBH_4_. The solution was mixed by inversion a few times. Immediately after the addition of NaBH_4_, the solution colour changed from yellow to light brown indicating the formation of 2–3 nm gold seed particles.

### Synthesis of Au NRs

First, a CTAB solution was prepared. Mixing of a CTAB solution is difficult at room temperature, therefore, the solution was heated to 45 °C under constant magnetic stirring until a clear solution was obtained. This solution was cooled down to room temperature before further processing. To the CTAB solution, various volumes of 0.1 M HAuCl_4_ stock solution were added to obtain different concentrations of HAuCl_4_ in different solutions. The solutions were mixed a few times by inversion. To these solutions, various amounts of 1 M HCl were added. This was followed by addition of various volumes of 0.1 M AgNO_3_. Subsequently, different volumes of 0.1 M AA solution were added. In each solution, the HAuCl_4_-to-AgNO_3_ molar ratio of 5 and the AA-to-HAuCl_4_ molar ratio of 1.4 was maintained. For example, the lowest HAuCl_4_ concentration of 0.5 mM in growth solution was obtained by adding 50 μl of 0.1 M HAuCl_4_ solution in 10 ml of CTAB solution. To this solution, 10 μl of 0.1 M AgNO_3_ and 70 μl of 0.1 M AA were added making the final concentration of AgNO_3_ and AA to be 0.1 and 0.7 mM, respectively.

With the addition of AA, the solution colour changes from yellowish to colourless. It should be noted that for lower HAuCl_4_ concentrations, the change in solution colour is fast, but for higher concentrations of HAuCl_4_, it takes more time for the colour of the solution to change. For example, for a HAuCl_4_ concentration of 0.5 mM, the change in colour with the addition of AA is almost instantaneous; while for 5 mM HAuCl_4_, it takes about 1 min for the colour to change.

Finally, various volumes of seed solutions were added for various HAuCl_4_ concentrations. For the 0.5 mM HAuCl_4_ concentration, 20 μl of seed solution was added. For the higher HAuCl_4_ concentrations, the added seed solution was increased in proportion to the HAuCl_4_ concentrations. This procedure ensures the same Au concentration per seed for all samples. The solution was left undisturbed at 26 °C overnight. The synthesis can be easily scaled up to 100 ml of growth solution volume.

For SEM imaging, all samples were centrifuged twice at 10,000 rpm and redispersed in distilled water. A 10-μl drop was dried on a Si substrate for imaging.

## Results and discussion

**Fig. 1:**
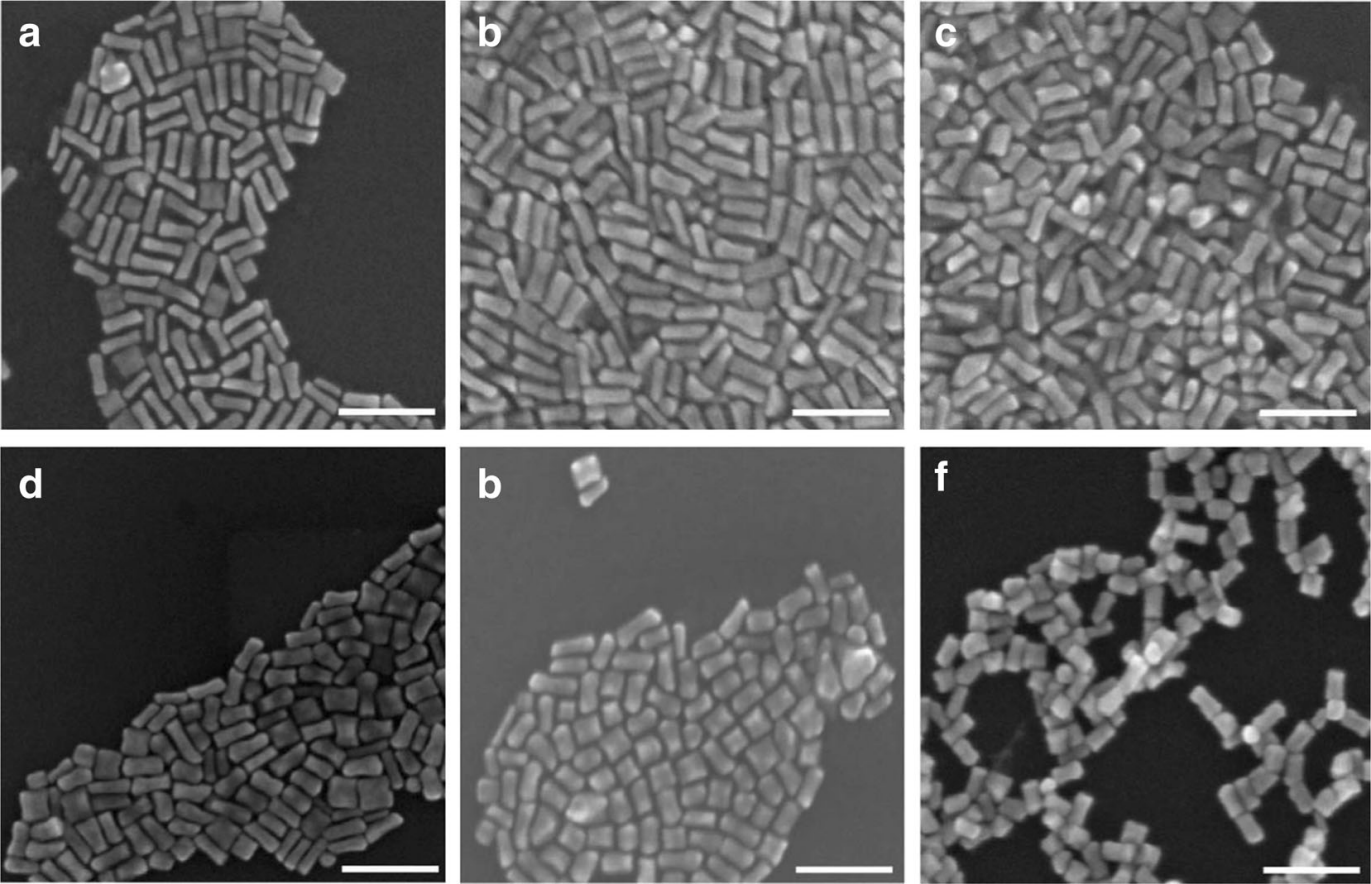
SEM images of NRs prepared with HAuCl_4_ concentrations of 0.5 (**a**), 0.65 (**b**), 0.8 (**c**), 1 (**d**), 2 (**e**) and 5 mM (**f**). *Scale bar* is 100 nm

**Fig. 2:**
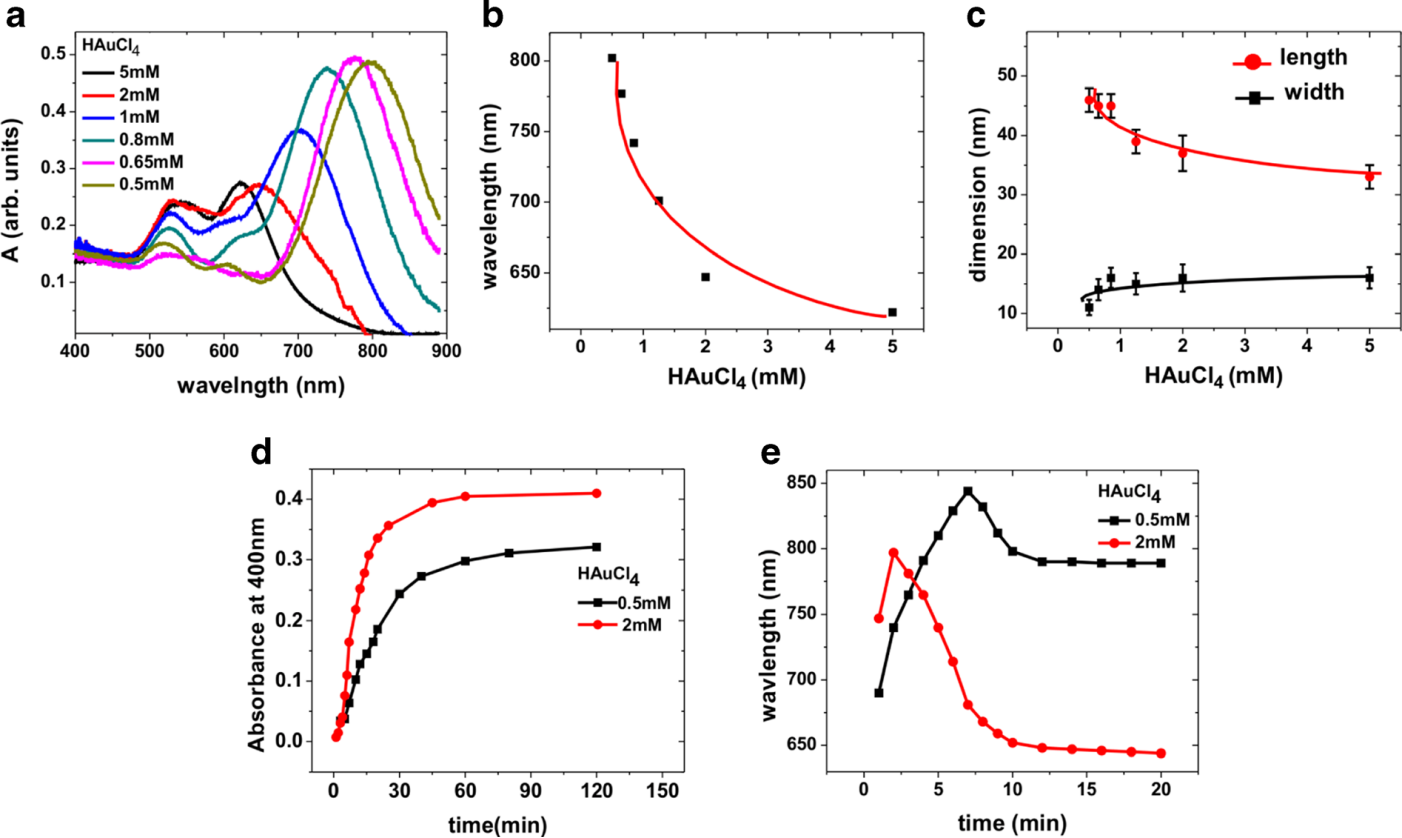
Observed variations in the properties of Au NRs for various HAuCl_4_ concentrations (without controlling the pH of the solution by HCl), as seen in the optical spectra (**a**) (the spectra are normalised at 400 nm to facilitate comparison); the position of the longitudinal plasmon resonance in the spectra as a function of HAuCl_4_ concentrations (**b**); the variation of the average length and width of the NRs as obtained from the SEM images as shown in Fig. 1 (**c**); the time evolution of the absorbance at 400 nm for 0.5 mM and 2 mm HAuCl_4_ (for 2 mM the absorbance values are divided by 4) (**d**); and variation of the position of the longitudinal plasmon resonance with time during the growth of NRs for 0.5 and 2 mM HAuCl_4_ (**e**). The *solid lines* in **b** and **c** are guides to the eye

Figure 1a–f depicts the SEM images of Au NRs synthesised with HAuCl_4_ concentrations of 0.5, 0.65, 0.8, 1, 2 and 5 mM, respectively. As described in the “Experimental methods,” the concentrations of AgNO_3_, AA and seeds varied in proportion to the HAuCl_4_ concentration, while the concentration of CTAB was kept constant at 0.1 M. Note that these samples were prepared without adding any HCl. As is evident from the SEM images (Fig. 1a–f) and the plot of Fig. 2c, there is a noticeable decrease in the aspect ratio of the NRs with an increase in Au concentration, resulting from a decrease in length as well as an increase in width of the NRs with increasing HAuCl_4_ concentrations.

The optical spectra of the NRs are shown in Fig. 2a. For all the samples, there are well-defined transverse (around 500 nm) and longitudinal (600–800 nm) plasmon peaks. Figure 2b depicts the variation of the longitudinal plasmon peak position with respect to the Au concentration in solution. A strong blue shift in longitudinal plasmon resonance is observed with increasing Au concentration for all samples. The blue shift indicates a decrease in length of the NRs (Kooij and Poelsema 2006) and is consistent with our observations from the SEM images.

Note that for all the samples, the ratios of Au, Ag, AA and seeds were kept constant. The only parameter changing was the effective CTAB concentration with respect to Au and Ag ions and AA and seeds. A higher concentration of the reactants and seeds in the growth solution can influence the growth kinetic in two ways. First, higher concentrations of Au(I) monomers and larger numbers of seeds are available in solution. Therefore, the monomers need to diffuse shorter distances to deposit on seeds during the growth process. Second, the selective surface passivation of the facets of the growing nanoparticles by CTAB, Br or AgBr, which are expected to be responsible for the anisotropic growth as proposed by various researchers (Nikoobakht and El-Sayed 2003; Gole and Murphy 2004; Liu and Sionnest 2005; Garg et al. 2010; Si et al. 2012; Grzelczak et al. 2008), is affected by a decrease in the molar ratio of CTAB with respect to Au, Ag and seeds. The overall result of this decrease in surface passivation and increase in diffusion flux is the formation of more isotropic shapes, i.e. NRs of lower aspect ratios.

In order to follow the effect of the reagent concentration on growth kinetics, we have measured the time evolution of the UV-Vis absorption spectra. The value of absorbance at 400 nm indicates the concentration of Au^0^ in the solution. Therefore, the time evolution of absorbance at 400 nm indicates the rate of NR growth. Figure 2d depicts the increase in absorbance at 400 nm with time, for samples prepared with Au concentrations of 0.5 and 2 mM. As there are four times more seeds for samples with 2 mM Au concentrations, the absorbance values for this sample are divided by 4 to obtain a better comparison. As evident from the figure, the rise in absorbance is considerably faster for samples prepared with 2 mM Au concentration. This clearly shows that the individual NRs are growing faster for samples prepared with higher Au concentrations.

Figure 2e shows the variation of the position of the longitudinal plasmon resonance as a function of growth time for the first 20 min of growth, for both samples. For 2 mM Au concentration, the maximum value for plasmon resonance wavelength (797 nm) is achieved within the first 2 min of growth. In comparison, it takes a growth time of 4.5 minutes for batches with 0.5 mM HAuCl_4_ to reach 797 nm. For this composition of the solution, the plasmon wavelength continues to increase and reaches a maximum value of 844 nm after a growth time of about 7 min. The maximum is followed by a sharp decrease in the plasmon peak position in both cases, with the resonance levelling off at a longer wavelength for the smaller concentration of HAuCl_4_.

The initial red shift for both samples is due to the increase in aspect ratio because of the increase in length at the start of the growth (Kooij and Poelsema 2006). The subsequent blue shift is a consequence of decrease in aspect ratio owing to the relatively faster increase in the width of the NRs. This is consistent with the results of previous studies of Au NR growth kinetics (Seo et al. 2009; Sau and Murphy 2004; Edgar et al. 2012). The faster increase in longitudinal plasmon wavelength for samples with higher Au concentration is due to the higher growth rate. As discussed before, the faster growth kinetics is due to the higher diffusion flux of monomers and the inefficient facet passivation of nanoparticles by AgBr/CTAB/Br.

The growth rate can be slowed down by lowering the pH of the reaction medium with the addition of HCl in the reaction medium. As the first pKa of AA is 4.1 at a lower pH value, only a small fraction of AA dissociates to ascorbate monoanions. It has been suggested that ascorbate is a stronger reducing agent compared to AA, and it accelerates the reduction of Au ions (Busbee et al. 2003; Ahmed and van Ruitenbeek 2016). Therefore, reduction of ascorbate anions by lowering the solution pH leads to a lower reduction rate and, hence, a slower growth rate of Au NRs.

**Fig. 3:**
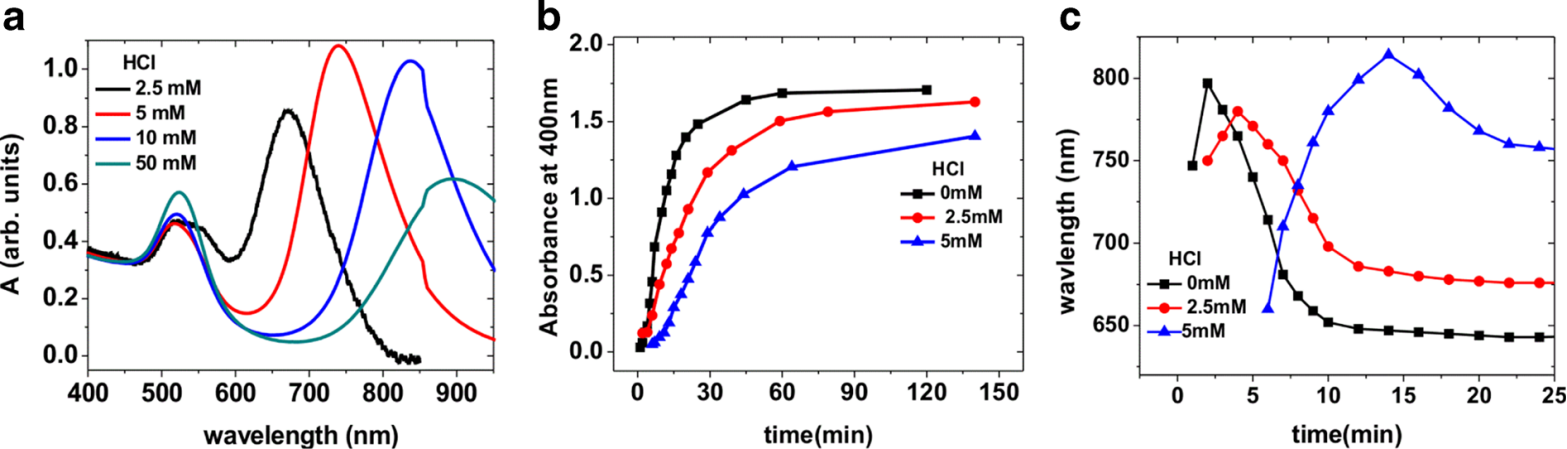
Optical spectra (**a**) of NRs prepared with a HAuCl_4_ concentration of 2 mM and various HCl concentrations. The spectra are normalised at 400 nm to facilitate comparison. Time evolution of the absorbance (**b**) at 400 nm for samples prepared with different HCl concentrations and for HAuCl_4_ concentration of 2 mM. Time evolution of the position of the longitudinal plasmon resonance (**c**) for NRs prepared with a HAuCl_4_ concentration of 2 mM and two different HCl concentrations

Figure 3 depicts the UV-Vis spectra of Au NRs synthesised with 2 mM HAuCl_4_ and with addition of various amounts of HCl. It is evident from the spectra that the longitudinal plasmon resonance red shifts with increase in HCl concentration. For addition of 2.5 mM HCl, the longitudinal plasmon peak is shifted to 670 nm, which represents a 25-nm red shift with respect to the sample prepared without HCl. There is a significant concentration of spherical nanoparticles nonetheless, which is evident from the presence of a broad transverse peak. The yield of NRs increases by increasing the amount of HCl to 5 mM in the growth solution. This is obvious from the narrowing of the transverse plasmon peak and from the longitudinal peak which red shifts to 740 nm, corresponding to a NR aspect ratio of nearly 3. Further increase in the HCl concentration leads to a further red shift of the longitudinal peak. However, there is a decrease in NR yield as is indicated by the increase in the transverse plasmon peak intensity. This suggests that there is an optimum concentration of HCl, necessary for high-yield synthesis of NRs of the desired aspect ratio.

Figure 3b shows the time evolution of the absorbance at 400 nm for NRs prepared with 2 mM HAuCl_4_ and different concentrations of HCl in growth solution. As evident from the figure, the rise in the absorbance value slows down with increasing HCl concentration in the reaction medium, which indicates slowing of growth rate with increasing HCl concentration. Figure 3c shows the time evolution of the longitudinal plasmon peak for samples for two different concentrations of HCl. While the position of the longitudinal resonance peaks after 2 min of reaction time without adding HCl (Fig. 1d), it reaches its maximum value much later, after 4 and 15 min of reaction time for 2.5 and 5 mM HCl, respectively. This clearly shows that the growth is slowed down by the presence of HCl in the growth medium. Furthermore, the higher the concentrations of HCl, the slower and more anisotropic is the growth.

**Fig. 4:**
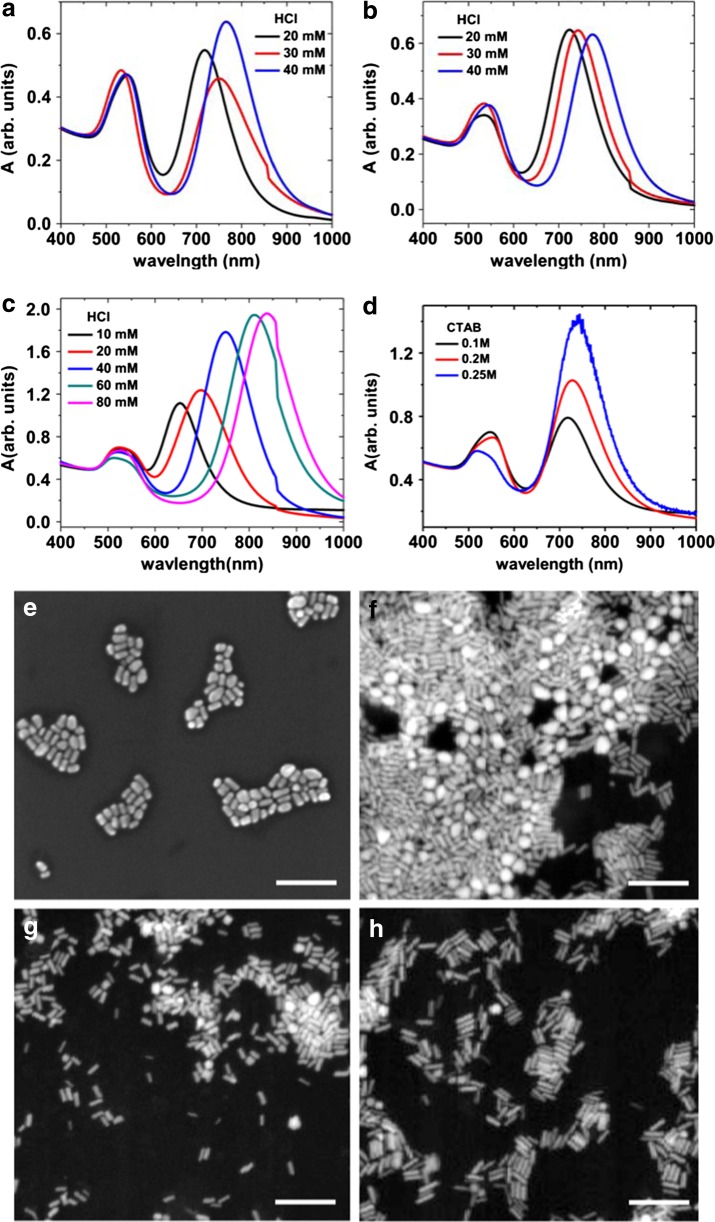
Optical spectra of NRs prepared with 5 mM HAuCl_4_ and various concentrations of HCl. The CTAB concentration is 0.1 (**a**), 0.2 (**b**) and 0.25 M (**c**). For comparison, we also show the optical spectra (**d**) for NRs prepared with 40 mM HNO_3_, 5 mM HAuCl_4_ and different concentrations of CTAB. All spectra are normalised at 400 nm for a better comparison. The lower panels show SEM images of Au NRs prepared with 5 mM HAuCl_4_ and CTAB = 0.1 M, HCl = 20 mM (**e**); CTAB = 0.2 M, HCl = 20 mM (**f**); CTAB = 0.25 M, HCl = 20 mM (**g**); and CTAB = 0.25 M, HCl = 60 mM (**h**). The *scale bar* is 200 nm

For still higher concentrations of HAuCl_4_, we find that the yield of NRs cannot be controlled simply by adjusting the concentration of HCl. Figure 4a shows the optical spectra of NRs prepared by using 5 mM HAuCl_4_ and 0.1 M CTAB in the growth solution. Although the longitudinal plasmon red shifts with increase of HCl concentration (Fig. 4a), the yield of NRs is low as observed from the significant intensity of transverse plasmon resonance peak around 530 nm, representing the presence of substantial amounts of nearly spherical nanoparticles. The presence of a fraction of spherical particles (about 45%) is confirmed by the SEM image in Fig. 4e.

The yield of NRs can be increased by increasing the concentration of CTAB in solution. Figure 4b depicts the UV-Vis spectra of Au NRs synthesised with 0.2 M CTAB and 5 mM HAuCl_4_. As evident from the spectra, there is a significant decrease in the transverse plasmon peak intensity which represents lowering of the spherical nanoparticle by-products. The longitudinal peak red shifts with increase of HCl concentration, which indicates that HCl is promoting the anisotropic growth. However, there is still a noticeable concentration of spherical nanoparticles in the final yield, as seen from the transverse plasmon peak. This is also evident from the SEM image (Fig. 4f), which indicates the presence of about 20% spherical nanoparticles for the sample prepared with 0.2 M CTAB and 20 mM HCl concentration.

We find that NRs in high yield (>90%) can be obtained by further increasing the CTAB concentration to 0.25 M. Figure 4c depicts the optical spectra of Au NRs synthesised with 5 mM HAuCl_4_, 0.25 M CTAB and various concentrations of HCl. There is a marked decrease in the transverse plasmon peak intensity in this case compared with NRs prepared with lower concentrations of CTAB (Fig. 4a, b), which indicates a higher yield of NRs. The SEM images of NRs prepared with 0.25 M CTAB (Fig. 4g, h) confirm that more than 90% of the particles are NRs, a much higher yield compared with the results for CTAB concentrations of 0.1 and 0.2 M (Fig. 4e, f).

For 0.25 M CTAB, NRs were prepared in the highest yield for addition of 60 mM HCl to the growth solution, which is evident from the diminishing transverse plasmon peak for this sample and from the SEM image of the NRs in Fig. 4g. Above and below this optimum HCl concentration, there is a slight decrease in yield. The average aspect ratio of the NRs increases with the increase of HCl concentration as shown by the red shift in the longitudinal plasmon resonance. It is important to note that, compared to the batches with lower concentrations of HAuCl_4_ (2 mM), higher concentrations of HCl are required to achieve a similarly large aspect ratio of the NRs. For example, in order to obtain a plasmon peak close to 750 nm, 40 mM HCl is needed in contrast to 5 mM HCl in the case of 2 mM HAuCl_4_. This agrees with our interpretation that for growth for 5 mM HAuCl_4_ is faster than that for 2 mM HAuCl_4_. Therefore, a higher concentration of HCl is needed for obtaining the optimum growth rate. Consequently, the HCl concentration in the reaction medium needs to be optimised for each HAuCl_4_ concentration (Table 1).

**Table 1:** Optimum HCl concentration for high yield synthesis of NRs with longitudinal plasmon peak around 800 nm for various HAuCl_4_ concentrations

HAuCl_4_ (mM)	HCl (mM)	CTAB (M)	Longitudinal peak position (nm)
0.5	0	0.1	804
1	1	0.1	817
2	10	0.1	839
5	60	0.25	809

From the results described previously, we also conclude that a CTAB-to-HAuCl_4_ molar ratio of 50 is required for optimum yield of NRs. It is well established that ligand exchange, i.e. conversion of [AuCl_4_]^−^ to [AuBr_4_]^−^, takes place when HAuCl_4_ is mixed with CTAB in solution (Pérez-Juste et al. 2004; Scarabelli et al. 2015). [AuBr_4_]^−^ subsequently forms a complex with the cation of the surfactant, [CTA][AuBr_4_]. This complex is insoluble in aqueous solutions unless the number of CTA-AuBr_4_ molecules per CTAB micelle is less than unity. The micelle aggregation number for CTAB is roughly 60 (Pérez-Juste et al. 2004; Scarabelli et al. 2015). Consequently, the HAuCl_4_-to-CTAB molar ratio should be close to 60 for the complete solubility of HAuCl_4_. This is close to the value of the CTAB-to-HAuCl_4_ molar ratio obtained by us for optimum yield of NRs.

An alternative interpretation for the role of HCl, rather than in controlling the pH, could be in supplying Cl^−^ ions that may replace the Br^−^ ions on the gold surface. To test for this possibility, Fig. 4d shows the results for NRs prepared with another acid, 40 mM HNO_3_, at the same 5 mM Au concentration and different concentrations of CTAB. As evident from the figure, the transverse plasmon peak is supressed with an increase in the CTAB concentration. These results are similar to those for the samples produced with HCl. Therefore, it is likely that the lower bound on the CTAB-to-HAuCl_4_ molar ratio, as seen in Fig. 4, comes from the solubility constraint of HAuCl_4_ in CTAB solution.

## Conclusion

In conclusion, we demonstrate that the yield of Au NRs can be increased in a seed-mediated method by controlling the pH of the reaction medium. Addition of an optimum concentration of HCl in the growth solution leads to slowing down of the growth kinetics and a high-yield synthesis of NRs of tuneable aspect ratios. Furthermore, we show that a CTAB-HAuCl_4_ molar ratio of about 50 is sufficient for high-yield synthesis of concentrated Au NRs. Our method will enable cost-effective and large-scale synthesis of Au NRs for a wide range of applications.
